# Exploring Opportunities to Leverage Pharmacists in Rural Areas to Promote Administration of Human Papillomavirus Vaccine

**DOI:** 10.5888/pcd17.190351

**Published:** 2020-03-12

**Authors:** Grace Ryan, Eliza Daly, Natoshia Askelson, Felicia Pieper, Laura Seegmiller, Tessa Allred

**Affiliations:** 1 University of Iowa College of Public Health, Iowa City, Iowa; 2 University of Iowa Public Policy Center, Iowa City, Iowa; 3 Iowa Cancer Consortium, Coralville, Iowa

## Abstract

Rural pharmacists have been identified as potential partners, along with health care providers, schools, and public health agencies, in administering and promoting the human papillomavirus (HPV) vaccine. We sought to understand the role of pharmacists in this work. We interviewed 11 pharmacists working at independently owned pharmacies in Iowa to explore their perspectives on HPV vaccine administration and promotion. Most pharmacists agreed that HPV vaccination was within their professional scope. They identified factors that facilitate vaccine administration (eg, accessibility of pharmacies). They also reported personal barriers (eg, lack of information, concerns about safety) and organizational barriers (eg, time and staff capacity). Future work should focus on alleviating barriers and building on strengths to improve vaccination rates and ultimately prevent HPV-related cancers.

**Summary**

**What is already known on this topic?**

Parents are willing to use pharmacies as an alternative to clinics for their children’s vaccination services, including the human papillomavirus (HPV) vaccine. However, the willingness of pharmacists to administer or promote this vaccine, especially in rural areas, is not well understood.

**What is added by this report?**

Rural pharmacists have the potential to be effective collaborators for HPV vaccine administration and promotion, but barriers, such as lack of education and capacity, exist.

**What are the implications for public health practice?**

Barriers to using pharmacists as providers of HPV vaccine could be overcome through partnerships with stakeholders who are already promoting the administration of HPV vaccine, and these barriers should be the focus of future research.

## Objective

Nationally, 51.1% of adolescents are up-to-date with the human papillomavirus (HPV) vaccine series (1). Rural populations are less likely to be vaccinated (2) and have higher rates of HPV-associated cancers (3). To increase HPV vaccination rates in rural areas, collaborations for vaccine administration and promotion should be explored. Pharmacists are potential partners, especially since 22 states, including Iowa, passed legislation permitting pharmacists to administer the HPV vaccine to adolescents (4). Previous research demonstrated high levels of acceptance in the United States of vaccinations administered by pharmacists (5,6). The objective of this study was to describe rural pharmacists’ role in administering and promoting the HPV vaccine in counties in Iowa with low rates of HPV vaccine uptake.

## Methods

As part of a larger study assessing HPV vaccination barriers and facilitators in rural counties with low HPV vaccination rates, we interviewed pharmacists at independently owned pharmacies in Iowa. We first identified 7 rural counties that had 1) HPV vaccination completion rates lower than the state average (27%), and 2) a percentage-point discrepancy larger than the average discrepancy in Iowa (31 percentage points) between completion rates for HPV vaccination and completion rates for other adolescent vaccinations (7). We used National Center for Health Statistics definitions of rurality and Iowa’s Immunization Registry for data on vaccination completion rates. We identified independently owned pharmacies (n = 14) through internet searches and conducted interviews in May and June 2018. We designed our interview guide by using questions and concepts adapted from previous projects (8,9) and included the following topics: the role of rural, independent pharmacists in HPV vaccine promotion and uptake; willingness to educate parents, refer patients, and administer the HPV vaccine; priority of HPV vaccine promotion; and vaccination barriers and facilitators in the pharmacy and the community (8,9). This project was determined not to be human subjects research by the University of Iowa Institutional Review Board. We attempted to contact 14 pharmacists by telephone up to 4 times to complete an interview and ultimately completed 11 interviews (average duration, 9.5 min). All interviews were audiorecorded and transcribed verbatim by a third-party service.

After an initial examination of transcripts, the research team created a codebook. Two researchers independently (E.A., W.B-B.) used it to code the same transcript, with additional codes to capture all relevant information. They met with a third researcher (G.R.) to resolve coding discrepancies and finalize the codebook. At this point, they determined that data saturation had been reached and remaining transcripts were divided for final coding. After all transcripts were coded, the research team met to discuss themes and subthemes that emerged from the codes.

## Results

Of the 11 pharmacists interviewed, 3 reported offering no vaccines and only 1 reported offering the HPV vaccine (Table 1). We identified 4 themes: pharmacists’ role in HPV vaccine administration and promotion, personal barriers to vaccine administration, organizational barriers to vaccine administration, and facilitators for vaccine administration (Table 2). Pharmacists reported that HPV vaccination should be a priority for adolescent health but that it was not a priority in their workplaces. Most indicated that recommending HPV vaccination was within their role. Many pharmacists were willing to educate and refer patients, but fewer reported willingness to administer the vaccine.

**Table 1:** Demographic Characteristics of Pharmacists (N = 11) Participating in Study on Using Pharmacists in Rural Areas to Promote Administration of Human Papillomavirus (HPV) Vaccination and Vaccinations Offered at Their Pharmacies, Iowa, May–June 2018

Characteristics	No. (%)
**Sex**	
Male	5 (45.5)
Female	6 (54.5)
**Vaccines offered**	
None	3 (27.3)
Hepatitis A	1 (9.1)
HPV/Gardasil	1 (9.1)
Influenza	7 (63.6)
Meningitis	1 (9.1)
Pneumococcal	7 (63.6)
Shingles	8 (72.7)
Tetanus, diphtheria, and pertussis	4 (36.4)

**Table 2:** Summary of Themes and Subthemes and Sample Quotes From Interviews of 11 Pharmacists Participating in Study on Using Pharmacists in Rural Areas to Promote Administration of Human Papillomavirus (HPV) Vaccination, Iowa, May–June 2018

Theme	Subtheme	Sample Quotes^a^
Pharmacists’ role in HPV vaccine administration and promotion	—	“Not all pharmacies may agree or be comfortable” (May 22, F.P., pharmacist A). “Oh, I’d be interested. I think educating people is a good idea, so there would be interest for sure” (May 2, F.P., pharmacist B). “I feel like [HPV vaccination] was just not a common thing that’s gonna come up in the pharmacy” (June 6, T.A., pharmacist C).
Personal barriers to vaccine administration	Sensitivity of subject	“Because of HPV and how you get HPV, I feel like sometimes it can be a sensitive subject” (May 22, F.P., pharmacist A).
	Lack of information	“The information continues to change [so] it’s always a matter of staying up to date with reading and following . . . resources. On that particular vaccine, I’m probably not as up to date as I should be” (June 6, F.P., pharmacist C). “I’m not familiar with the costs of it all, the storage, those kind of things” (June 14, T.A., pharmacist D).
	Concerns about safety	“I have a few concerns just with the HPV hype about injury and things that have happened to people after they’ve gotten [it], like back pain” (June 6, T.A., pharmacist E).
	Misinformation	“Yes, it’s considered a rural area, but I think in general we have good coverage” (June 11, T.A., pharmacist F). “Medicaid does not allow us to do it for those under 18, even with a prescription” (June 20, T.A., pharmacist G). “[Adolescents] are supposed to go to the doctor’s office” (June 4, T.A., pharmacist H).
Organizational barriers to vaccine administration	Time and staff capacity	“Usually there’s only one pharmacist . . . so that interrupts everything to do the vaccination” (June 11, T.A., pharmacist F).
	Liability	“You incur a little bit more liability when you’re dealing particularly with an adolescent, because generally they have a greater risk of fainting or having an episode after a vaccination” (June 6, F.P., pharmacist C).
	Competition with local health care providers	“I feel that we probably need to work more closely with the clinics to be more collaborative with the clinics, so that they didn’t feel like anybody was stepping on anyone’s toes” (June 6, F.P., pharmacist C).
Facilitators for vaccine administration	Accessibility	“As far as in our rural community, customers or patients are very likely to pop in the pharmacy and ask questions. We’re very accessible, whereas a practitioner really isn’t. So, I think it’s just the ease of availability of information for them” (June 4, T.A., pharmacist H).
	Increase in advertising	“If they knew it was available at a pharmacy . . . you know how they’ve got Facebook and everything, stuff can spread pretty fast” (June 4, T.A., pharmacist H).

a Each quote is followed by the date of the interview, the initials of the interviewer, and an identifier for the pharmacist interviewed.

We identified 4 subthemes for personal barriers to HPV vaccine administration: sensitivity of subject, lack of information, concerns about safety, and misinformation. Pharmacists reported insufficient knowledge to recommend, refer, or educate parents about the vaccine. Although no pharmacists cited religious or moral objections, some reported that discussion of the vaccine could have a political, and therefore contentious, aspect.

Organizational barriers to HPV vaccination administration were time and staff capacity, liability, and competition with local health care providers. Although some pharmacists reported that they were not certified to administer the vaccine, others had not created protocols for administering the vaccine. Lack of space, such as consultation rooms big enough for both a parent and an adolescent, was also described as a barrier. Barriers cited less frequently were related to liability in administering the vaccine, low numbers of adolescents coming to the pharmacy, and the potential to be seen as competitive with local health care providers.

Pharmacists also identified 2 factors that could facilitate administration and promotion of the vaccine: accessibility of community pharmacies and an increase in advertising through social media. Many recognized the better accessibility and convenient hours of pharmacies, compared with clinics, for busy parents. Other potential facilitators were more training to increase knowledge about the vaccine and how to administer it and collaborating with health care providers, schools, or public health agencies.

## Discussion

Our aim was to better understand the role of independent pharmacists in administering and promoting the HPV vaccine in rural Iowa. Overall, we found that although barriers exist to HPV vaccine administration and promotion, most pharmacists interviewed were willing to overcome them with support and training. Similar to our study, recent studies also identified time, staff constraints, and lack of integration with clinics as barriers to HPV vaccine administration and promotion (10,11). Also similar to other studies, our study indicated that participants identified convenience and accessibility as facilitators for vaccine administration and promotion (12).

However, many pharmacists expressed a need for more information and training on the vaccine and how to administer it. More information and training could overcome barriers they identified. Given their interaction with adolescents and parents, pharmacists could not only provide HPV vaccinations but also act as a vaccine champion by educating parents, distributing information, and providing referrals to local health care providers. Finally, although a few expressed concerns about being viewed as competition for clinics, others saw opportunities for valuable partnerships with clinics to establish referral systems or combine promotion or advertising activities. 

A limitation of our study is the small sample of 7 rural counties. Our results should not be generalized beyond this setting.

Ultimately, our findings offer insight into the potential to work with pharmacists to increase HPV vaccine uptake in rural areas. This work should focus on simultaneously overcoming barriers while using the strengths noted by these pharmacists (ie, accessibility and convenience). Partnerships between pharmacists and state public health agencies or academic institutions could be explored as one pathway toward overcoming barriers (13). Effectively supporting pharmacists in administering and promoting HPV vaccination will translate to increased HPV vaccination rates and prevention of future HPV-related cancers.
